# ApoE lipoproteins in the central nervous system under homeostasis and role in Alzheimer’s disease and related disorders

**DOI:** 10.1186/s13024-026-00970-0

**Published:** 2026-07-07

**Authors:** Michael R. Strickland, David M. Holtzman

**Affiliations:** 1 Department of Pathology and Immunology, Washington University in Saint Louis, St. Louis, MO 63110 USA; 2 Department of Neurology, Washington University in Saint Louis, St. Louis, MO 63110 USA; 3 Hope Center for Neurological Disorders, Washington University in Saint Louis, St. Louis, MO 63110 USA; 4 Knight Alzheimer’s Disease Research Center, Washington University in Saint Louis, St. Louis, MO 63108 USA

**Keywords:** Apolipoprotein, Apolipoprotein E, Alzheimer’s disease, Lipoprotein, Central nervous system

## Abstract

Apolipoprotein E (ApoE) is highly expressed in the central nervous system (CNS) where it plays a critical role in lipid homeostasis and in the etiology of Alzheimer’s disease (AD) and related diseases. ApoE associates with lipids to form discoidal and spherical lipoproteins that carry lipid and protein cargo throughout the brain. In this review, we focus on the significance of ApoE as a lipoprotein and how this impacts the function of ApoE in homeostasis and disease. In the CNS, ApoE is primarily secreted by astrocytes, though other cells, including microglia, secrete ApoE under certain conditions. ApoE lipoproteins (LpE) secreted by different cell types carry unique lipids and proteins which alter its function. The lipidation state of ApoE alters its conformation and binding to different receptors and, consequently, its ultimate impact on AD pathology. Most dramatically, nonlipidated ApoE has minimal binding to the low density lipoprotein receptor (LDLR), while lipidation of ApoE restores high affinity binding to LDLR. Furthermore, the degree of ApoE lipidation also impacts ApoE receptor binding through changes in protein conformation and stoichiometry of ApoE molecules per lipoprotein. The lipidation state of ApoE also alters its interaction with amyloid-β and tau, the proteins involved in forming amyloid plaques and neurofibrillary tangles, the pathological hallmarks of AD. LpE also carry lipids and proteins that alter the function of ApoE. Understanding how the lipid and protein content of LpE interacts with the conformational changes that occur with lipidation and maturation are essential to mechanistically understanding the role of ApoE in homeostasis and disease pathogenesis. In this review, we highlight the current understanding of LpE biology in the CNS and delineate important areas of future research.

## Background

Apolipoprotein E (ApoE) is the most abundant apolipoprotein in the central nervous system [1], followed by ApoA1 and ApoJ [2]. ApoE is thought to function as a shuttle for cholesterol and other lipids throughout the brain which relies on the de novo synthesis of cholesterol rather than dietary cholesterol [3]. The brain is the most cholesterol rich organ of the body and thus proper lipid handling and delivery is critical, especially in the setting of central nervous system injury and disease. ApoE has received much interest due to the ε4 allele of the *APOE* gene (which encodes the ApoE4 protein) dramatically increasing Alzheimer’s disease (AD) risk. A single copy of the ε4 allele increases disease risk by approximately four-fold while two copies of the allele increase disease risk by approximately twelvefold relative to individuals who are ε3/ε3 [1, 4, 5]. In contrast to ε4, the presence of the ε2 allele is protective relative to individuals who are ε3/ε3 [4, 6]. ApoE is 299 amino acids long and the only amino acid differences between ApoE2, ApoE3, and ApoE4 are at positions 112 and 158: ApoE2 cysteine/cysteine; ApoE3 cysteine/arginine; ApoE4 arginine/arginine. Many reviews have characterized ApoE and its role in homeostasis and impact on AD and AD pathology [1, 3, 4, 7]. In this review, we focus on the characteristics of ApoE as a lipoprotein and how differential lipidation and cargo shape its interactions with its receptors, cells, and ultimately contributes to disease pathogenesis.

ApoE is present in high density lipoprotein (HDL)-like particles in the central nervous system. Though similar in density to plasma HDL, ApoE lipoproteins (LpE) tend to be larger than those found in plasma, and although ApoE is present in the HDL fraction of plasma, HDL is typically associated with ApoA1. For clarity, we use the nomenclature LpE to refer to ApoE containing lipoproteins similar in density to HDL, especially prominent in CSF, in line with previous literature [8, 9]. In human CSF, there is a wide diversity of lipoproteins in the CSF with ApoE being the most abundant apolipoprotein. In addition to ApoE: ApoJ, ApoA1, ApoA2, and ApoA4 are major structural apolipoproteins in the CSF; ApoH and ApoD are also present in human CSF but are not classical apolipoproteins with amphipathic α-helical structure [8–10]. ApoE is primarily found alone but also associates with the other apolipoproteins to form hybrid particles. As will be discussed in greater detail later in this review, the lipidation state of ApoE alters its interactions with amyloid-β (Aβ), tau, and ApoE receptors. LpE carries additional protein and lipid cargo. This cargo differs based on the cellular origin of ApoE and ApoE isoform [11]. Different isoforms of ApoE are correlated with differences in cholesterol esters and other lipids. These lipids can dramatically alter the consequence of ApoE uptake by cells shifting ApoE from a protective to a detrimental phenotype. The ability of ApoE to efflux lipids from microglia also plays a significant role in disease pathogenesis as lipid loaded microglia are less able to contribute to tissue repair [12]. In this review we will examine the secretion through the secretory pathway and lipidation by ATP-binding cassette transporter ABCA1 (ABCA1); the lipidated conformation and role of enzymes in LpE maturation; how the cellular source of ApoE alters the lipid and protein cargo of ApoE; and how ApoE isoform alters the lipidome and proteome of LpE with discussion of the consequences for both homeostasis and disease pathogenesis.

### ApoE secretion and lipidation

**Fig. 1:**
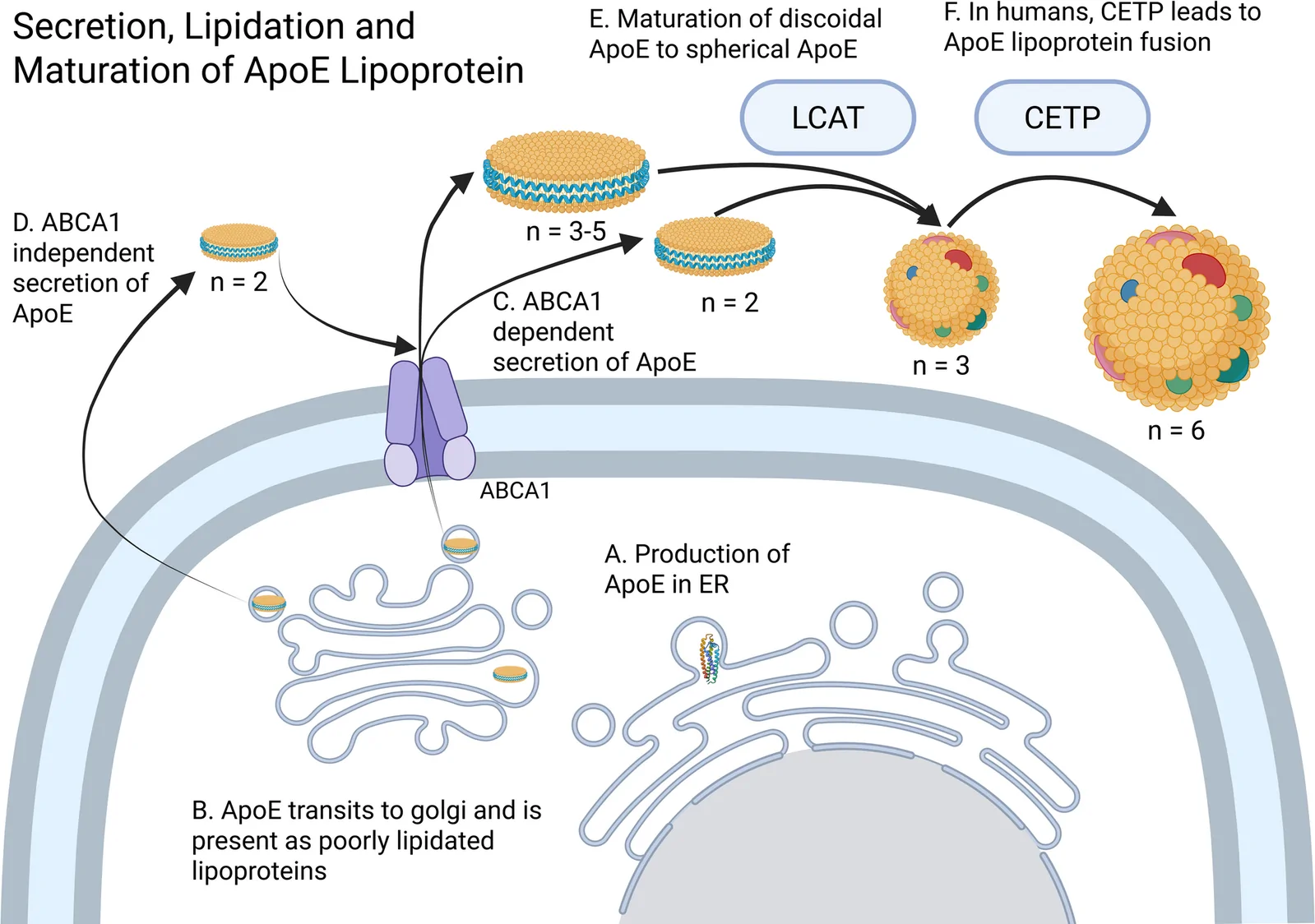
Secretion, lipidation, and maturation of ApoE lipoprotein. **A**. ApoE is produced in the endoplasmic reticulum prior to trafficking to the golgi. **B**. ApoE transits to the golgi complex and is lipidated by intracellular lipid transporters and lipidated ApoE is also present from recycling ApoE. This forms a pool of intracellular ApoE that can either be degraded or be secreted by the cell. **C**. ApoE can be secreted as small, poorly lipidated lipoprotein from the cell in an ABCA1 independent manner. ApoE secretion from this pathway can be stimulated by the ApoA1 and contributes to cholesterol efflux. **D**. The majority of ApoE is secreted through an ABCA1 dependent pathway. Knockout of ABCA1 dramatically reduces cellular secretion of ApoE and CSF levels of ApoE. ApoE is initially secreted as discoidal lipoprotein. Disks ranging in diameter from 8.7 to 14.5 nm contain two ApoE molecules per particle. Disks ranging in diameter from 16.2 to 28.2 nm can contain three to five ApoE molecules per particle. **E**. LCAT esterifies cholesterol in nascent LpE converting discoidal LpE to spherical LpE. Spherical LpE formed by the action of LCAT ranges from 10.3 to 11.5 nm in diameter and contains three ApoE molecules per particle. **F**. CETP, which is expressed in humans but not rodents, produces large spherical particles as the result of the fusion of LpE resulting in spherical particles ranging from 13.2 to 15.1 nm in diameter containing six ApoE molecules per particle

ApoE is produced for systemic circulation throughout the body by hepatocytes in the liver where approximately 70% of ApoE is present in very low density lipoproteins (VLDL) while approximately 30% is present in HDL [13]. ApoE is thought to be incorporated into both VLDL and HDL during secretion by hepatocytes [14]. However, human liver transplant studies show that systemic ApoE does not enter into the central nervous system due to the blood brain barrier and knockout of hepatic ApoE does not have an effect on Aβ pathology in the APP/PS1-21 mouse model of Aβ amyloidosis [15, 16]. Thus, all of the ApoE present in the central nervous system is produced de novo. Under homeostatic conditions, ApoE is primarily produced by astrocytes which secrete lipidated ApoE [17–20]. ApoE is secreted through the classical secretory pathway whereby it is produced in the endoplasmic reticulum (Fig. 1A), transits to the golgi and trans-golgi (Fig. 1B), and then is transported to the plasma membrane [21]. As part of the secretion pathway, ApoE is routed to the golgi where it is glycosylated and sialylated [22, 23]. ApoE then either is routed towards secretion or degradation. ApoE secretion occurs primarily through an ABCA1-dependent pathway (Fig. 1C). Knockout of ABCA1 in macrophages and astrocytes decreases the total amount of secreted ApoE and what is secreted is poorly lipidated [24–28]. This is also demonstrated in macrophages isolated from Tangier disease patients, caused by mutations that disrupt ABCA1 function, which have sever hyperplasia of the golgi network indicative of a secretion defect [29]. ApoE can also be secreted in an ABCA1-independent (Fig. 1D) manner following stimulation by exogenous ApoA1 or ApoE [24]. The initial lipidation of ApoE likely occurs intracellularly and discoidal ApoE has been isolated from hepatocyte golgi in the HDL fraction [30]. In astrocytes, ABCA1 was found to associate with ApoE in caveolin-1 rich domains supportive of intracellular lipidation of ApoE [31]. However, this interpretation is complicated by the fact that secreted ApoE is taken back up and transits through the recycling endosome pathway in multiple cell types, including astrocytes [32–36]. Following secretion, ApoE is retained at the plasma membrane surface due to association with heparin sulfate proteoglycans (HSPGs) and ApoE receptors, the proportion varying greatly by cell type [37, 38]. In the case of ApoA1, it has been proposed that further, or even initial lipidation, occurs at the cell surface via ABCA1 [39]. It is likely that ApoE is further lipidated following secretion through interaction with ABCA1, although further research is required to delineate the mechanisms underlying ApoE lipidation.

**Fig. 2:**
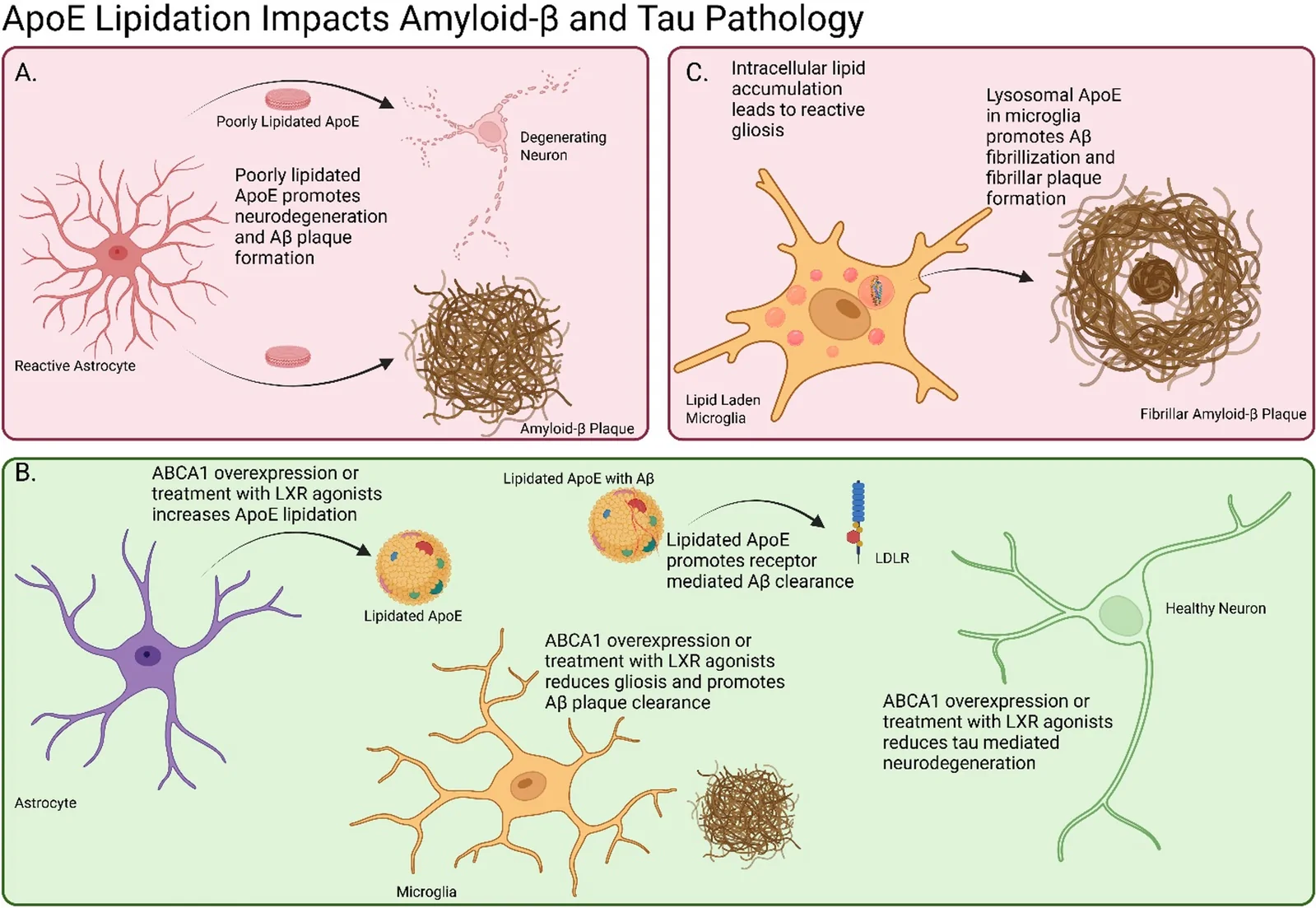
ApoE lipidation impacts amyloid-β and tau pathology. **A**. Reactive astrocytes secrete ApoE containing inflammatory lipids that lead to neuronal death. In vivo, astrocyte ApoE promotes Aβ plaque formation and tau-mediated neurodegeneration in mouse models of Aβ amyloidosis and mouse models of frontotemporal dementia. **B**. Overexpression of ABCA1 by transgene expression or treatment with LXR agonists leads to the secretion of more lipidated ApoE. ABCA1 overexpression leads to reduced gliosis and lipid accumulation. ABCA1 overexpression leads to reduced Aβ plaques through improved clearance of Aβ plaques and receptor mediated clearance of soluble Aβ. ABCA1 overexpression reduces tau mediated neurodegeneration and leads to a decrease in neuronal tau accumulation. **C**. Microglia becomes lipid laden and reactive in the context of Aβ and tau pathology. Lysosomal ApoE interacts with Aβ in lysosomes to promote Aβ fibrillization and the formation of fibrillar Aβ plaques and neuritic dystrophy

Knockout of ABCA1 in mice leads to the secretion of poorly lipidated ApoE by astrocytes between 7 and 8 nm in diameter, compared to particles with a diameter between 8 and 20 nm that are secreted when ABCA1 is present [25]. Global knockout of ABCA1 in mice results in levels of apoE that are decreased by 96% in plasma and 98% in cerebrospinal fluid (CSF), partly due to more rapid degradation following secretion [25]. The lipidation status of ApoE is an underappreciated component of the impact of ApoE on disease pathogenesis. Modulating the lipidation of ApoE results in significant effects on both amyloid and tau pathology. In the context of Aβ pathology, knockout of ABCA1 led to an increase in Aβ plaques (Fig. 2A) [40–42]. In contrast, overexpression of ABCA1 led to a decrease in Aβ plaques (Fig. 2B) [43]. The decrease in plaques is likely a result of either improved clearance of Aβ or the fact that poorly or nonlipidated ApoE is associated with greater Aβ seeding and aggregation rather than lipidated ApoE (Fig. 2B) [44]. Nonlipidated ApoE that is present in microglial lysosomes has been shown to result in ApoE aggregation and seeding of Aβ, resulting in Aβ plaque formation suggesting that more lipidated ApoE is resilient to aggregation and decreased seeding of plaques (Fig. 2C) [45].

Similar to other cells, ABCA1 expression is mediated by the transcription factors liver X receptor (LXR) and retinoid X receptor (RXR) that form a heterodimer to promote secretion of ApoE from astrocytes [46]. Pharmacologically altering ABCA1 expression using an LXR agonist has been shown to ameliorate Aβ and tau pathology [47, 48]. In the context of Aβ pathology exacerbated by high fat diet, treatment of APP23 mice on a high fat diet with an LXR agonist, T0901317, reduced amyloid pathology and raised CSF ApoE levels [48]. LXR agonist treatment has also been shown to be beneficial in reducing Aβ pathology in the context of traumatic brain injury [49]. Treatment with LXR agonist reduced neurodegeneration associated with tau and ApoE4 mediated neurodegeneration, reduced hyperphosphorylated tau accumulation, and lipid accumulation [47]. Currently, use of LXR agonists is complicated by side effects induced by activation of LXR/RXR in the liver causing hepatic steatosis and hypertriglyceridemia [50, 51]. However, development LXR agonists that avoid these side effects and are brain penetrant hold potential therapeutic promise for treating Alzheimer’s disease.

### Conformation of lipidated ApoE

In this review, we will briefly review the literature concerning the structure of LpE, which is of particular relevance to the structure of ApoE in the CNS. For more in depth reviews of ApoE structure, please refer to the following literature [4, 7, 52]. Early studies have characterized the structure of nonlipidated ApoE revealing that the nonlipidated N-terminal half of ApoE adopts a compact four-helix bundle [53]. However, this compact structure unfolds upon lipidation with unfolding of the four-helix bundle and ApoE adopting an extended conformation [54]. This conformational change allows ApoE to adopt a conformation suitable for binding receptors like LDLR [55]. Generation of discoidal ApoE using recombinant protein and lipidation with lipids such as 1-palmitoyl-2-oleoyl-glycero-3-phosphocholine (POPC) and dimyristoylphosphatidylcholine (DMPC) result in the formation of discoidal lipoprotein similar in diameter and conformation to astrocyte secreted human LpE [20, 56]. ApoE formed using the sodium cholate dialysis method forms particles ranging from 8 to 15 nm [17, 56]. Astrocytes secrete ApoE in a similar size ranging from 8 to 20 nm [17, 19, 20]. Studies using X-ray crystallography of dipalmitoylphosphatidylcholine (DPPC) lipidated ApoE revealed that ApoE was present in a spheroidal particle and proposed a “horseshoe” conformation of ApoE [57, 58]. In this model, the helical domains self-associate and form a semicircular arc around the spheroidal particle. However, resolution of this model was limited to around 10Å. It is also unclear if the crystallization procedure altered the structure of the lipoprotein as a result of the crystal packing. Alternatively, this may represent a different conformation of ApoE than that found in truly discoidal particles like those secreted by astrocytes. As DPPC lipidation results in small particles, typically of the size referred to as “lipid-poor” ApoE, this conformation may reflect nascent ApoE prior to further lipidation by ABCA1 [17, 25, 57, 58]. Recent results using cryo-electron microscopy in combination with monoclonal Fab and F’(ab)_2_ fragments showed that ApoE is present in a discoidal particles [17]. The binding of Fab and F’(ab)_2_ fragments to ApoE on discoidal particles suggest that the ApoE proteins are in an antiparallel conformation similar to the conformation described for apolipoprotein A1 (ApoA1) [17, 59, 60]. However, the cryoEM studies also resulted in a low resolution model preventing detailed molecular modeling of the alpha-helical structure of ApoE. A computational model combined with crosslinking mass spectrometry has also proposed a hairpin model for ApoE4 in discoidal particles [61]. In this model, the C-terminal domain of ApoE wraps around the disk with the N-terminal domain present as either a 4-helix bundle or an “open” extended conformation where the helix bundle is partially unfolded. The computational model of ApoE4 in discoidal particles is consistent with two ApoE being present in an antiparallel orientation similar to that described in the cryoEM model. It is important to note that in contrast to LpE in rodent CSF, human CSF-LpE are predominantly present as 11–24 nm spherical particles, therefore understanding the structural changes that occur when ApoE is present in spherical lipoprotein will be essential to understanding its role in vivo [62–64]. Evidence from ApoA1, a related apolipoprotein, also suggests that the degree of lipidation of discoidal ApoA1 alters its conformation [59]. This is potentially applicable to ApoE and therefore the degree of lipidation would be expected to alter ApoE structure in both discoidal and spherical lipoproteins. Another caveat in interpreting structural studies of recombinant ApoE is the use of synthetic and purified lipids for lipidation. These simplified systems are essential for understanding the biochemistry of ApoE, however, it should be noted that the addition of lipids, such as cholesterol, can modify the membrane properties of the lipoprotein. This is exemplified by cholesterol which undergoes a phase shift at low temperatures where it adopts a liquid crystal-like phase and has been shown to modify the structure of LDL lipoprotein [65]. Future work to determine a high resolution structure of ApoE will be necessary to distinguish and reconcile the proposed models of lipid bound ApoE.

**Fig. 3:**
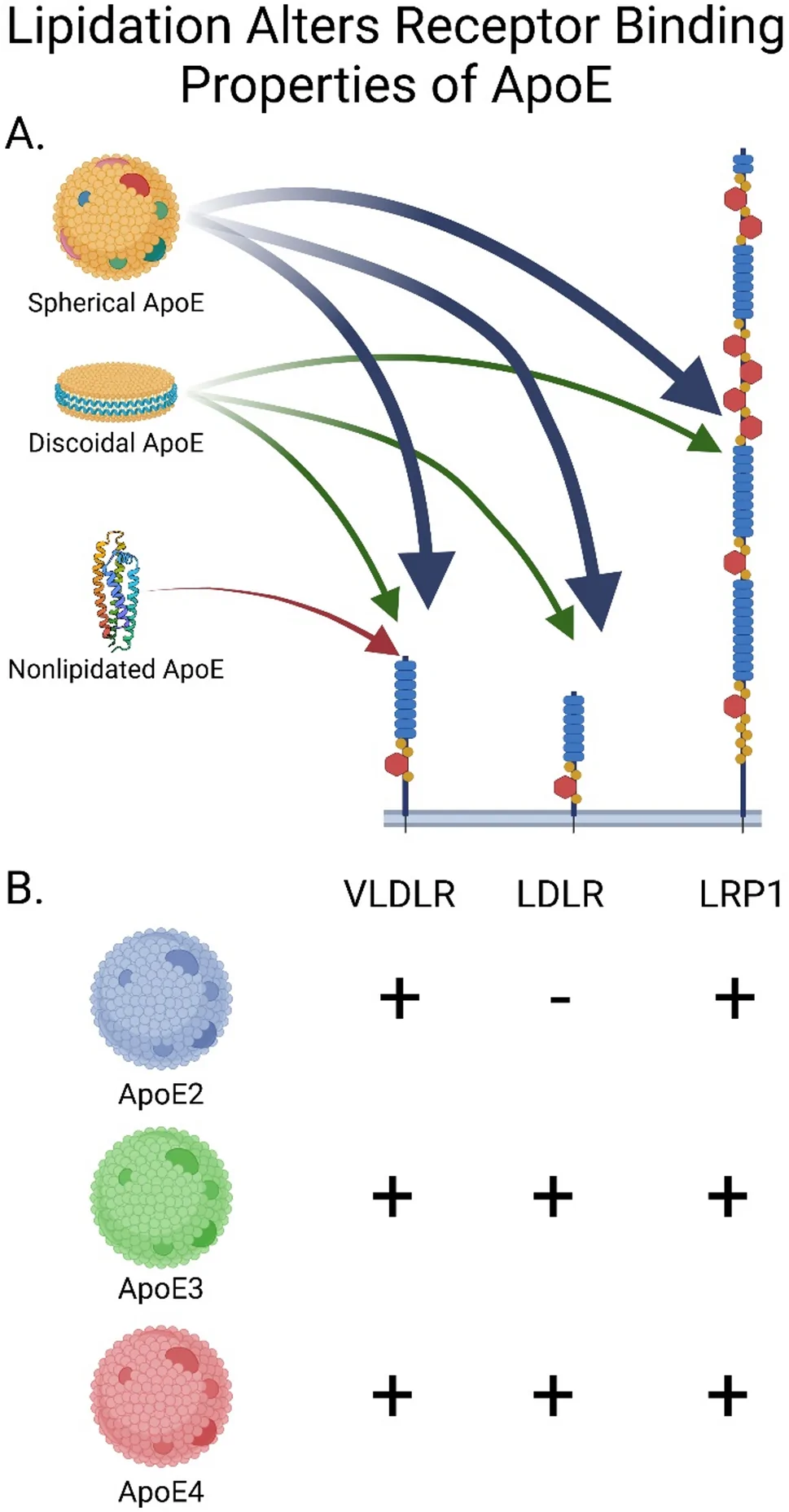
Lipidation alters receptor binding properties of ApoE. **A**. Nonlipidated ApoE, with arrows in red, binds VLDLR but has low affinity binding to LDLR and LRP1. Lipidation of ApoE allows for binding of ApoE to VLDLR, LDLR, and LRP1, arrows shown in green. Larger LpE containing more ApoE molecules per particle has higher avidity binding to ApoE receptors; spherical particles typically contain more ApoE protein per particle than discoidal particles, arrows shown in blue. **B**. Lipidated ApoE2 is unable to bind LDLR but is able to bind both VLDLR and LRP1. Lipidated ApoE3 and ApoE4 are able to bind VLDLR, LDLR, and LRP1

The lipidation state has important implications for binding of ApoE to different receptors and may drive preferential receptor binding, depending on the degree of ApoE lipidation (Fig. 3). Previous research has shown that nonlipidated ApoE is unable to bind LDLR [55, 66]. In addition, ApoE2 has dramatically reduced binding to LDLR even when lipidated [55, 66, 67]. As a consequence, ApoE2 is associated with type III hyperlipidemia and the accumulation of lipids in plasma as a result of impaired ApoE remnant clearance [68, 69]. Although a risk factor for type III hyperlipidemia, ApoE2 is protective for Alzheimer’s disease [6]. However, in contrast to LDLR, the different lipidated ApoE isoforms bind with similar affinity to low density lipoprotein receptor-related protein 1 (LRP1) [66]. Although lipidation is required for binding to both LRP1 and LDLR, nonlipidated ApoE is able to bind with high affinity to very low density lipoprotein receptor (VLDLR) [66]. VLDLR binds all ApoE isoforms with similar affinity. The degree and type of lipidation also impacts binding of ApoE to its receptors. Comparing recombinant ApoE lipidated with either DPPC or DMPC showed that particles made with DMPC had higher affinity to LDLR than particles made with DPPC [57].

In addition to conformational changes that occur when ApoE is lipidated, the size of LpE also alters the binding avidity to its receptors. This is primarily a stoichiometric effect whereby larger lipoprotein particles are able to accommodate more ApoE protein per particle (Table 1). This leads to a stepwise increase in LpE affinity with the number of ApoE proteins per particle [57]. For illustration, the increased binding of ApoE to LDLR compared to LDL is attributable to stoichiometry. An elegant study using different ratios of active vs. receptor inactive ApoE showed a step wise decrease in avidity with every inactive form of ApoE added [70]. This would suggest that nascent ApoE secreted by astrocytes which typically contain two ApoE per particle would have lower avidity to LDLR than more mature particles [17, 57]. Maturation of ApoE secreted from astrocytes from discoidal to spherical particles depends on cholesterol esterification by lecithin–cholesterol acyltransferase (LCAT), which is also secreted by astrocytes (Fig. 1E) [71]. In vitro studies using ApoE isolated from human patients was lipidated with POPC and unesterified cholesterol mixed with LCAT revealed that cholesterol esterification resulted in spherical particles [72]. Discoidal LpE from 10 to 14 nm in diameter contained ~ 2 ApoE protein per particle, while larger discoidal particles ~ 24–27 nm contained ~ 4–5 ApoE molecules per particle [72]. Treatment with LCAT resulted in spherical particles containing esterified cholesterol ranging from ~ 11–14 nm in diameter [72]. Another study incubating discoidal ApoE containing two ApoE protein per particle from 8.7 to 10.8 nm in diameter with LCAT found that they formed spherical particles ~ 10.4 nm in diameter with a predicted 3 ApoE molecules per particle based on the diameter measured by electron microscopy [56]. Similarly, incubation of discoidal ApoE with a diameter of 17 nm with LCAT resulted in spherical particles ~ 11.4 nm in diameter with 3 ApoE molecules per particle as measured by crosslinking [73]. Although LCAT plays a role in LpE maturation, knockout of LCAT has been reported to have minimal to no impact on Aβ pathology in a mouse model of amyloidosis [74]. However, due to the impact of ApoE lipidation on receptor binding and the induction of lipofuscinosis by injection of polyunsaturated fatty acid - cholesterol esters (PUFA-CEs) in the context of tau pathology, it would be of interest to study the impact of LCAT metabolism in the context of tauopathy and tau-mediated neurodegeneration [75]. Furthermore, LCAT activity has been reported to be significantly reduced in the CSF of AD patients compared to control patients [76, 77].

**Table 1:** Relationship between lipoprotein size, conformation, and stoichiometry

Lipoprotein Conformation	Treatment	Diameter (nm)	ApoE Molecules per Lipoprotein	Reference
Discoidal	None	8.7–14.5	2	[17, 56, 72, 118]
Discoidal	None	16.2–28.2	3–5	[17, 72, 118]
Spherical	LCAT	10.3–11.5	3	[56, 73]
Spherical	CETP	13.2–15.1	6	[73]

The number of ApoE molecules that can be accomodated per lipoprotien varies due to the conformation of the lipoprotein particle (discoidal or spherical), the size of the lipoprotein, and modification by enzymes such as LCAT and CETP

Cholesteryl ester transfer protein (CETP) is an enzyme expressed in humans, but not in rats, mice, or canines, that alters the lipidation of CSF ApoE (Fig. 1F). Studies in humans, which express CETP, reveal that CSF-LpE is primarily present as spherical particles rather than the primarily discoidal particles observed in rodent and canine CSF [9, 64]. Incubation of spherical ApoE with CETP results in the generation of larger spherical particles due to particle fusion [78]. These larger particles that are formed due to particle fusion are predicted to contain 6 ApoE molecules per particle with a diameter ~ 13–15 nm and thus would also be predicted to have higher avidity to ApoE receptors. However, the significance of CETP in AD pathogenesis is unclear. Several studies investigating known CETP polymorphisms have shown no impact on AD risk [79, 80]. However, when assessing AD risk associated with the ApoE4 allele, CETP polymorphisms such as the C-629 A and Taq1B polymorphisms are associated with decreased AD risk [80, 81]. In a recent large study of atherosclerotic cardiovascular disease, the CETP inhibitor obicetrapib significantly attenuated increases in the marker for brain amyloidosis p-tau217 compared to placebo [82]. Further study of the role of CETP is warranted as it may explain some differences in human lipoprotein metabolism compared to rodent and canine models as well as being a promising therapeutic target for AD.

LpE also impact AD disease pathogenesis through association with Aβ and tau. Larger LpE secreted by astrocytes, those with a diameter of 10–17 nm, were found to associate with Aβ [18]. Aβ has also been reported to associate with ApoE specifically, in contrast to ApoJ containing lipoprotein for instance, in human CSF [83, 84]. However, another study using ultracentrifugation isolation of LpE found very little association between monomeric Aβ and lipidated ApoE [85]. However, Aβ oligomers were found to associate with poorly lipidated ApoE rather than lipidated ApoE [85]. Clearance of Aβ through direct association with ApoE or through binding to ApoE receptors decreases amyloid pathology in mouse models. Overexpression of LDLR increased amyloid-β clearance, reduced ApoE levels, and decreased Aβ pathology [86]. Similarly, knockout of LRP1 in astrocytes decreases Aβ clearance and increases Aβ pathology [85, 87]. However, this effect may be cell type specific as knockout of LRP1 in neurons resulted in reduced amyloid pathology [88, 89]. LpE may also compete with tau for uptake by ApoE receptors. Overexpression of the ApoE receptor LDLR in the brain decreases tau mediated neurodegeneration [90]. Mutations in ApoE that reduce binding to LDLR, namely ApoE2, but also to HSPG and LRP1, like the APOE Christchurch variant, also decrease tau seeding in wildtype mice and in the context of Aβ pathology [91, 92].

### Cell type specific secretion of ApoE lipoprotein and contribution to Alzheimer’s disease

In the central nervous system (CNS), ApoE is primarily secreted by astrocytes under homeostatic conditions with microglia upregulating ApoE secretion under multiple disease conditions [18–20, 93–95]. Knockout of ApoE in astrocytes versus microglia has differing impacts on disease pathogenesis in mouse models of Aβ amyloidosis suggesting that ApoE produced from different cell types may differentially affect disease outcomes [96, 97]. Knockout of human ApoE3 and ApoE4 in astrocytes of transgenic mice results in markedly decreased Aβ plaque pathology despite microglia continuing to produce ApoE [96]. Microglial specific knockout of murine apoE does not alter amyloid-β pathology in contrast to astrocyte specific ApoE knockout [97]; though decreasing microglia ApoE4 modestly decreased Aβ deposition [98]. Total body apoE knockout as well as astrocyte specific ApoE knockout results in markedly decreased fibrillar amyloid deposition and in less compact Aβ plaques as well as decreasing neuronal loss and reactive gliosis in a tauopathy model [96, 99, 100]. Although there are very few true amyloid plaques in the absence of mouse apoE, there is greater neuritic dystrophy around the remaining plaques [96, 99]. In light of the results from the Simons group that apoE associates with Aβ in microglial lysosomes [45], combined with the findings modulating astrocyte ApoE, suggest that astrocytic apoE seeds the initial stages of Aβ plaque formation in microglial lysosomes followed by the release into the extracellular space of seeds that form fibrillar plaques. Over time uptake of plaque material by microglia results in further plaque compaction.

**Fig. 4:**
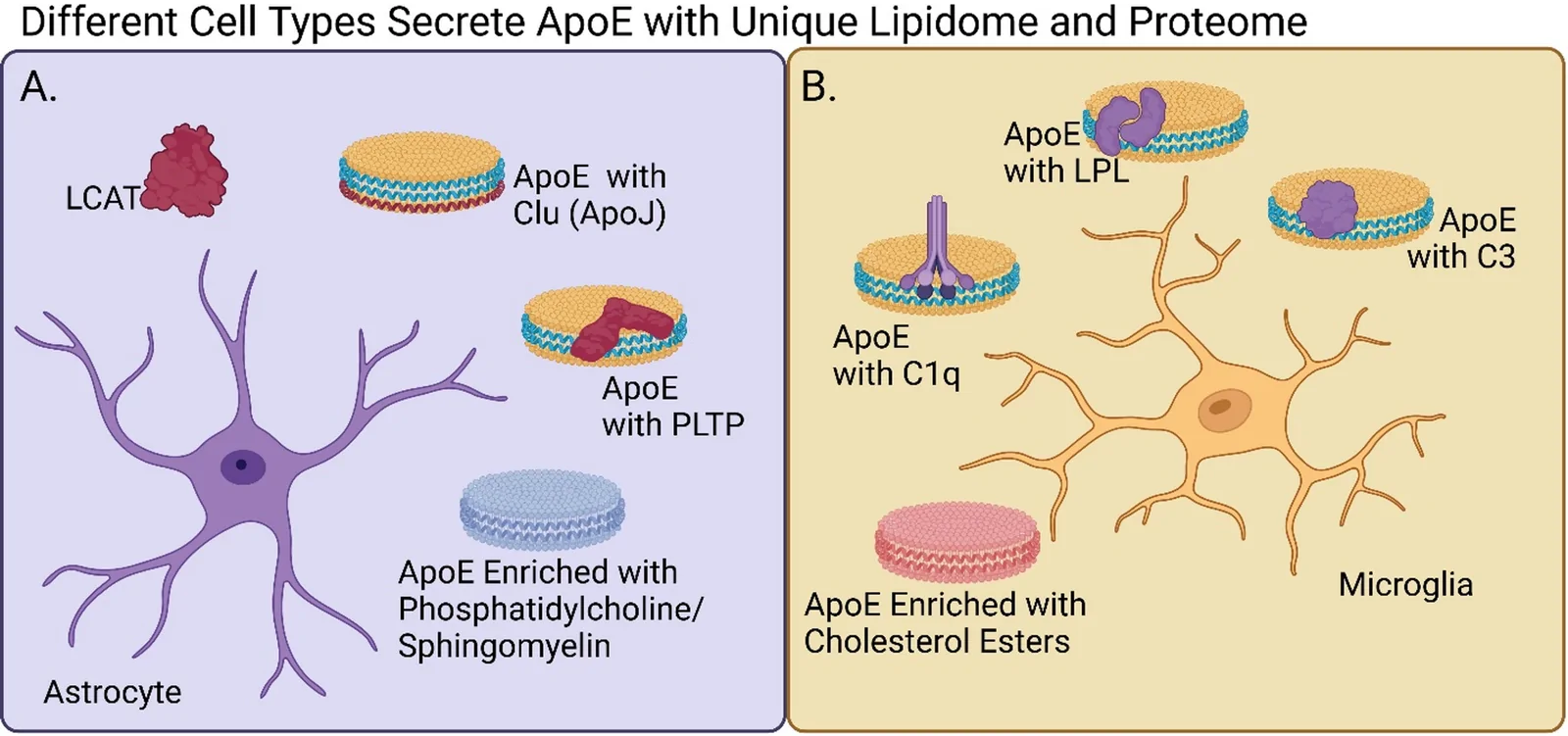
Different cell types secrete ApoE with unique lipidome and proteome. **A**. Astrocytes secrete LCAT into the extracellular space which promotes the formation of spherical ApoE. Astrocyte secreted LpE is enriched in Clu (ApoJ) and PLTP. Astrocyte ApoE is enriched in phosphatidylcholine compared to microglial ApoE. Astrocyte ApoE is associated with Aβ plaque formation and neurodegeneration. **B**. Microglia secreted ApoE is enriched with C1q, LPL, and C3. Microglia secreted LpE is enriched in cholesterol esters relative to astrocyte ApoE. Microglia engulfment of Aβ leads to association with nonlipidated ApoE in lysosomes and the formation of fibrillar Aβ plaques

Another factor that could contribute to the differing impact of astrocyte and microglial secreted LpE could be the lipid and proteome content of LpE (Fig. 4). Astrocytic LpE was found to be enriched in proteins such as phospholipid transfer protein (PLTP) and clusterin (Clu, also known as ApoJ), both of which play a role in AD disease pathogenesis (Fig. 4A) [11, 19, 101]. In human CSF, PLTP was also shown to be present on LpE based on immunoaffinity purification of ApoE [101]. PLTP, which is reported to transfer phospholipids to HDL in plasma, has reduced activity in human AD patient CSF [101, 102]. Clu, which is secreted separately from ApoE-containing lipoproteins but can be found together with ApoE in CSF lipoproteins, was also identified as a risk factor for AD in a genome wide association study [19, 103, 104]. Cultured microglial secreted LpE was found to be associated with complement component 1q (C1q) and complement component 3 (C3), proteins involved in the complement cascade pathway (Fig. 4B) [11]. ApoE binding to C1q was found to attenuate inflammation through high affinity binding to the C1q complex where it interrupts the complement cascade [105]. Knockout of the complement component 3a receptor 1 (C3aR1) in PS19 mice, a model of tauopathy, results in attenuated inflammation and decreased neurodegeneration suggesting a potential role for ApoE to mitigate tau-mediated neurodegeneration. However, the impact of ApoE on tau mediated neurodegeneration is likely strongly dependent on the lipidation status of ApoE. Treatment of PS19 mice with an LXR agonist reduces tau-mediated neurodegeneration [47]. Further, global knockout of ApoE also very strongly suppresses neurodegeneration and knockout of APOE4 from astrocytes also strongly attenuates tau-mediated degeneration [92, 106]. Treatment of cultured astrocytes with an LXR agonist reduced the cholesterol ester content of astrocyte secreted LpE [11], which has been reported to drive lipofuscinosis in cells that uptake LpE-containing PUFA-CEs [75]. Further research characterizing the lipidome and proteome content of astrocyte and microglia secreted LpE in different disease contexts will be necessary to disentangle the contributions of astrocyte and microglial ApoE to AD and related disorders.

In addition to astrocytes and microglia, other cell types in the brain have been reported to produce ApoE under different conditions. These cells include oligodendrocytes, pericytes, vascular smooth muscle cells, perivascular fibroblasts, and perivascular macrophages [107–109]. Neurons have also been reported to express low levels of ApoE mRNA and knockout of ApoE4 under control of the synapsin-1 promoter results in decreased tau-mediated neurodegeneration [110]. Expression of ApoE4 in oligodendrocytes has been reported to lead to cholesterol dysregulation, namely accumulation of cholesterol esters and reduced axonal myelination by oligodendrocytes [111]. Pericytes have been shown to secrete ApoE similar in size to astrocyte secreted LpE but containing higher cholesterol levels than astrocyte secreted ApoE [112]. Pericyte upregulation of ApoE is associated with Aβ accumulation along vessels in an in vitro blood brain barrier model [113]. Vascular smooth muscle cells and peri-vascular fibroblasts also express ApoE and upregulate ApoE expression when quiescent [114, 115]. Perivascular macrophages express ApoE and play a role in neurovascular health and injury [116]. Expression of ApoE4 specifically in perivascular macrophages increases white matter damage in a model of cerebral hypoperfusion [116]. How the lipidome and proteome of LpE secreted by these different cell types differ and the impact on AD pathogenesis remains to be explored.

### Impact of ApoE isoform on lipidation, proteome, lipidome, and consequences for homeostasis and Alzheimer’s disease pathogenesis 

ApoE isoform has also been reported to alter LpE lipid and protein content. Lipidation of recombinant, human ApoE isoforms result in discoidal particles of similar size and conformation [17, 117]. Astrocytes with human *APOE* isoforms knocked into the endogenous mouse *Apoe* allele secrete discoidal particles of overlapping size. However, ApoE2 lipoproteins secreted from astrocytes form slightly larger particles than ApoE3 and ApoE4 lipoproteins [17–20]. Given the impact that LpE size potentially has on receptor binding, this may drive preferential isoform receptor interaction independent of the effects of the ApoE2 cysteine at amino acid position 158. Larger particles are also able to contain a higher stoichiometric ratio of ApoE to lipid which may also influence receptor binding and interaction with enzymes like LCAT [56, 118]. When ApoE is secreted from astrocytes or recombinantly lipidated, ApoE adopts an antiparallel dimer of the surface of lipidated particles [17]. This is similar to ApoA1 in HDL which adopts an antiparallel dimer [60, 119]. The alignment of the two ApoA1 proteins in discoidal particles alter the ability of ApoA1 to activate LCAT and alters the rate of cholesterol esterification [119]. ApoE3 has been reported to form homodimers in human plasma and in the CNS [120, 121]. The disulfide linkage of ApoE3 on LpE reduces the affinity for ApoE3 lipoprotein to LDLR [121]. It is intriguing to hypothesize that the disulfide linkage forming between ApoE3 on lipoprotein particles is locking ApoE3 into a registry with lower binding affinity to LDLR. It may also explain how treatment of ApoE2 which forms more dimers and binds very poorly to LDLR, following treatment with reducing agents such as cysteamine, has partially restored binding to LDLR [122, 123]. Future work is necessary to elucidate whether ApoE registry alters receptor binding and would have important implications for mechanistically understanding how ApoE isoform impacts receptor binding.

ApoE isoforms have also been reported to influence the lipid composition of LpE. Comparing the ApoE isoforms, Lindner et al. found that ApoE4 bound more to phosphoinositide (PIP)-containing liposomes [124]. After loading astrocytes with oleic acid, the group also found that ApoE4 lipoproteins contained more TAG rich particles compared to ApoE2 and ApoE3 astrocytes [124]. Assessing microglia secreted LpE, ApoE4 lipoproteins were found to contain lower lipid amounts than particles secreted by ApoE3 microglia [125]. ApoE isoform also alters the proteome of microglial secreted LpE. Microglia secreted ApoE4 lipoprotein contains higher levels of lipoprotein lipase (LPL) and C1q than ApoE2 and ApoE3 microglia secreted lipoproteins [11]. ApoE4 expressing microglia have been reported to be more inflammatory possibly due to reduced cholesterol efflux; therefore, association of ApoE with C1q secreted by microglia may be a compensatory mechanism to decrease inflammation. Additionally, LPL secretion by macrophages has been reported to increase lipid accumulation and inflammation in the context of atherosclerosis [126]. ApoE4 is known to cause dramatic lipid dysregulation in microglia under certain conditions that is associated with altered lipid transport, accumulation of triglycerides and cholesterol esters, and accumulation of lipid droplets [127]. ApoE4 expressing microglia have also been reported to accumulate larger lipid droplets and secrete more proinflammatory cytokines in response to lipopolysaccharide (LPS) stimulation [128]. Lipid droplet containing microglia have been associated with the *APOE ε4/ε4* genotype in human AD brain [129]. Microglia conditioned media, derived from lipid droplet containing human iPSC-derived microglia, added to iPS neurons resulted in tau phosphorylation and apoptosis [129]. In mouse astrocytes expressing human ApoE, ApoE has been reported to directly associate with lipid droplets [130]. ApoE4 was found to lead to disruption in the structure of lipid droplets in astrocytes and cause higher levels of lipid peroxidation due to higher levels of unsaturated triglycerides in lipid droplets [130]. In the context of tauopathy, disruption of lipid droplet formation in microglia was found to increase the accumulation of endosomal/lysosomal lipids and exacerbate neurodegeneration in the PS19 mouse model of tau-mediated neurodegeneration [131]. Future work will be needed to elucidate the role of ApoE in lipid droplets in vivo and in the context of AD pathology.

ApoE isoform also impacts the interaction of ApoE with other apolipoproteins. Apolipoprotein C1 (ApoC1) was found to be present at higher levels in ApoE2 and ApoE3 microglia secreted lipoproteins and is reported to inhibit binding of ApoE to LRP1 and LDLR [132]. This result is mirrored in human CSF where ApoC1 was found to be significantly reduced in APOE ε4 carriers compared to ApoE ε3/ε3 homozygotes [133]. ApoC1 has been reported to reduce TNFα and IL-6 secretion in mouse and human macrophages [133]. In contrast to total ApoC1 protein levels, the proportion of truncated proteolytic fragments of ApoC1 and ApoC2 were elevated in the CSF of human APOE ε4 carriers [134]. Aβ42 levels are negatively correlated to these truncated forms suggesting greater plaque deposition [134]. The ApoC1 gamma haplotype has also been found to increase AD risk independent of ApoE [135]. Apolipoprotein A2 (ApoA2) has been found to form a disulfide linked heterodimer with ApoE2 and ApoE3 [121, 136]. The ApoE-ApoA2 complex is predominant in the HDL fraction of ApoE in plasma and the ApoE-ApoA2 heterodimer is more stable under ultracentrifugation conditions [137]. The ApoE-ApoA2 heterodimer has also been isolated in human CSF samples [63, 138]. The ApoE-ApoA2 heterodimer has lower affinity to LDLR than ApoE alone [137]. Since ApoE4 does not contain any Cys residues and cannot form a heterodimer with ApoA2, this may have implications for the higher rate of ApoE4 clearance from plasma compared to ApoE3 [139]. Apolipoprotein D (ApoD) was found to be enriched in astrocyte secreted LpE [11]. ApoD levels increase in AD though this increase appears to be limited to ε3/3 carriers compared to ε4/4 carriers [140, 141]. In plasma, ApoE has not been reported to form a heterodimer with ApoD, however, whether this occurs in the CNS is currently unclear [142].

## Future directions

Understanding how LpE composition alters the interactions of ApoE with its receptors and pathological proteins will be critical for mechanistically understanding the role of ApoE in homeostasis and disease pathogenesis. The lipidation state of ApoE dramatically alters its binding affinity to different receptors due to both conformational changes in ApoE and the number of ApoE molecules per particle. Understanding how to modulate ApoE lipidation through drugs such as LXR agonists may yield therapies for Alzheimer’s disease and similar neurodegenerative diseases. An important area of research that has been gaining more attention is how ApoE isoforms alter the lipids carried by LpE that can have neurotoxic effects [143] or lead to microglial lipid accumulation [75]. Further research should further explore how disease conditions alter the lipidome of LpE and how this potentially alters receptor interactions leading to differential cell uptake. Another area of interest is the protein cargo of LpE. LpE cargo differs based on the cellular source leading to differential impacts of ApoE on other cells [11]. This includes complexes of ApoE with other apolipoproteins which alters the receptor binding properties of LpE. Understanding how different protein cargo alters the functionality of LpE will be critical in mechanistically understanding how ApoE secreted from different cell types has disparate impacts on homeostasis and disease pathogenesis. Finally, determining a high resolution structure of ApoE in discoidal and spherical LpE will provide novel insights into its normal function and in the pathophysiology of disease.

## Conclusion

ApoE plays critical roles in homeostasis and in the etiology of Alzheimer’s disease. Although this review has focused on the consequences of LpE primarily in the context of AD, ApoE also has profound roles in the development of diseases such as cardiovascular disease, macular degeneration, and cancer. ApoE is one of the exchangeable apolipoproteins and is in the same gene family as ApoA1 [144, 145]. Similar to other exchangeable lipoproteins, ApoE is dependent on ABCA1 for lipidation and efficient secretion, although some ApoE is secreted in an ABCA1 independent manner. The lipidation of ApoE dramatically alters its function determining its interaction with ApoE receptors and pathological proteins such as Aβ and tau. Poorly lipidated ApoE has diminished affinity to LDLR and LRP1 and is more prone to associate with Aβ oligomers and promote Aβ plaque formation. Treatment of mice with LXR agonists which upregulate the expression of ABCA1 lead to the secretion of more lipidated ApoE. This has been shown to have a beneficial impact on both Aβ and tau pathology; suggesting a potential therapeutic avenue for the treatment of AD and related tauopathies. Complicating the picture even more are differences in the lipid and protein composition of ApoE secreted by different cell types and under conditions of lipid stress. Carefully disentangling the impact of ApoE isoform, lipid composition, and protein cargo of LpE from different cell types under homeostatic and disease conditions will be critical in developing a mechanistic understanding of ApoE function. This is especially important in understanding the pleiotropic effects of ApoE, especially ApoE4, on disease progression [146]. In this review we have pulled together literature addressing the mechanics of ApoE lipidation, the secretion of LpE by different cell types, the impact of ApoE lipidation on its receptor interactions, and the impact of ApoE isoform on the lipidome and proteome of LpE. Advances in mass spectrometry and cryogenic electron microscopy now offer a chance to characterize the structure, lipidome, and proteome of LpE in unprecedented depth. These insights will provide a mechanistic basis to understand LpE function. We look forward to future research that will help to answer how LpE functions to maintain lipid homeostasis and lead to the development of AD with an eye to translating these results into much needed therapies.

## Data Availability

No datasets were generated or analysed during the current study.

## Abbreviations

ABCA1: ATP-binding cassette transporter ABCA1
AD: Alzheimer’s disease
ApoA1: Apolipoprotein A1
ApoA2: Apolipoprotein A2
ApoC1: Apolipoprotein C1
ApoD: Apolipoprotein D
ApoE: Apolipoprotein E
Aβ: Amyloid-β
C1q: complement component 1q
C3: Complement component 3
C3aR1: Complement component 3a receptor 1
CE: Cholesterol ester
CETP: Cholesterol ester transfer protein
Clu (ApoJ): Clusterin (Apolipoprotein J)
CNS: Central nervous system
CSF: Cerebrospinal fluid
DMPC: Dimyristoylphosphatidylcholine
DPPC: Dipalmitoylphosphatidylcholine
HDL: High density lipoprotein
HSPG: Heparan sulfate proteoglycan
LCAT: Lecithin–cholesterol acyltransferase
LDL: Low Density Lipoprotein
LDLR: Low Density Lipoprotein Receptor
LPL: Lipoprotein lipase
LPS: Lipopolysaccharide
LRP1: Low density lipoprotein receptor-related protein 1
LXR: Liver X receptor
PIP: Phosphoinositide
PLTP: Phospholipid transfer protein
POPC: 1-palmitoyl-2-oleoyl-glycero-3-phosphocholine
PUFA: Polyunsaturated fatty acid
RXR: Retinoid X receptor
VLDL: Very low density lipoprotein
VLDLR: Very low density lipoprotein receptor
